# Complete Genome Sequence of a SARS-CoV-2 Strain Isolated in Northern Germany

**DOI:** 10.1128/MRA.00520-20

**Published:** 2020-06-04

**Authors:** Susanne Pfefferle, Jiabin Huang, Dominik Nörz, Daniela Indenbirken, Marc Lütgehetmann, Lisa Oestereich, Thomas Günther, Adam Grundhoff, Martin Aepfelbacher, Nicole Fischer

**Affiliations:** a Institute for Medical Microbiology, Virology, and Hygiene, University Medical Center Hamburg-Eppendorf, Hamburg, Germany; b Heinrich Pette Institute, Leibniz Institute for Experimental Virology, Hamburg, Germany; c Bernhard Nocht Institute, Leibniz Institute for Tropical Medicine, Hamburg, Germany; Queens College

## Abstract

Here, we describe the complete genome sequence of a severe acute respiratory syndrome coronavirus 2 (SARS-CoV-2) strain isolated from an oropharyngeal swab sample from a female patient with COVID-19 who was infected in Hamburg, northern Germany.

## ANNOUNCEMENT

A novel coronavirus, severe acute respiratory syndrome coronavirus 2 (SARS-CoV-2), was first identified in Wuhan, China, as the alleged cause of a cluster of severe pneumonia cases (1). The virus has since spread worldwide, affecting more than 3.6 million people and causing more than 250,000 deaths by 5 May 2020; the disease was named COVID-19. Taxonomic classification placed the novel virus within the subgenus *Sarbecovirus*, genus *Betacoronavirus*, family *Coronaviridae*, order *Nidovirales* (2).

Here, we describe the full-genome sequence of a SARS-CoV-2 strain (SARS-CoV-2/human/DEU/HH-1/2020) isolated from a female patient with COVID-19. The infection was acquired in Hamburg, northern Germany. All studies were carried out in keeping with local legal and regulatory requirements. Written informed consent was obtained from the patient. Also, approval by the local ethics committee (Ethik-Kommission Ärztekammer Hamburg) was obtained (approval number PV7306).

The isolate was obtained from an upper respiratory tract specimen (oropharyngeal swab) from a 62-year-old woman who was part of a small local cluster of COVID-19 cases, originating from a household contact who likely acquired the virus while traveling in Italy. The patient herself did not show any signs or symptoms at the time the sample was taken (3). The swab sample (ESwab; Copan, Italy) tested SARS-CoV-2 RNA positive at the Institute for Microbiology, Virology, and Hygiene at the University Medical Center Hamburg-Eppendorf by real-time reverse transcription-PCR (RT-PCR), as described previously (4). For virus isolation, 500 μl of the remaining (not inactivated) ESwab medium was used for adsorption on Vero cells (ATCC CRL-1586) seeded in T25 flasks. After 1 h at 37°C, the ESwab medium was replaced by cell culture medium (Dulbecco’s modified Eagle’s medium containing 3% fetal calf serum, 1% penicillin-streptomycin, 1% l-glutamine [200 mM], 1% sodium pyruvate, and 1% nonessential amino acids [all from Gibco/Thermo Fisher Scientific, Waltham, MA, USA]). Cells were monitored daily for cytopathic effect (CPE). No CPE was observed until day 4, but virus growth was confirmed by real-time RT-PCR. Additional experiments revealed that infection of different Vero cells (ATCC CCL-81) resulted in a strong CPE at ∼24 to 48 h postinfection.

For sequencing of the viral RNA, nucleic acid extraction was performed automatically using the QIAsymphony DSP virus/pathogen kit and 200 μl cell culture supernatant (supplemented 1:1 with Roche PCR medium for inactivation). An RNA Illumina next-generation sequencing (NGS) library was prepared from the sample using the SMARTer stranded total RNA-Seq kit v2-pico input mammalian (TaKaRa Bio Europe, Saint-Germain-en-Laye, France). The library was multiplexed and sequenced (10 samples were included) on an Illumina NextSeq instrument according to the manufacturer’s protocol.

A total of 18,971,906 paired-end 76-nucleotide (nt) reads (2,883,729,712 bp) were generated by the Illumina NextSeq sequencer. Bases with a score of less than Q30, as well as adapter sequences of the reads, were trimmed and any reads shorter than 35 nt were removed using Trimmomatic v0.36 (5). The samples after trimming contained 18,369,904 high-quality paired-end reads, with 61,312 × 2 reads (e.g., 122,624 reads) mapping to the reference Wuhan-Hu-1 sequence (GenBank accession number NC_045512.2) (6). The mapped reads were used as input data for the SPAdes assembler (v3.13.0) (7), resulting in a single, positive-stranded RNA of 29,870 bp, with no gaps and a mean coverage depth of 289.1×. There were no missing bases in the untranslated regions (UTRs), in either the 5′ UTR or the 3′ UTR [excluding the poly(A) tail], compared with the reference genome. The Prokaryotic Genome Annotation Pipeline (PGAP) (8) identified 10 putative open reading frames (ORFs) in the putative complete genome of the HH-1 isolate. The variants were called with the tool freebayes (9). As shown in Table 1, a total of five single-nucleotide polymorphisms and one multinucleotide polymorphism were detected. Overall, the genome of the isolate HH-1 has 99.97% nucleotide identity with the reference Wuhan-Hu-1 genome (as analyzed with the software EMBOSS:6.6.0.0 water), suggesting a high degree of similarity between them. Default parameters were used for all software unless otherwise specified.

**TABLE 1:** Nucleotide variants of the SARS-CoV2 sequence of the HH-1 isolate, in comparison to the reference strain (GenBank accession number NC_045512.2)

Nucleotide position	Reference nucleotide(s)	HH-1 nucleotide(s)	Variant type	Nucleotide change^*a*^	Amino acid change (position)	Gene name
160 (noncoding)	G	A	Upstream gene variant	NA	NA	NA
241 (noncoding)	C	T	Upstream gene variant	NA	NA	NA
3037	C	T	Synonymous variant	ttC/ttT	Phe to Phe (924)	ORF1ab
14408	C	T	Missense variant	cCt/cTt	Pro to Leu (4705)	ORF1ab
23403	A	G	Missense variant	gAt/gGt	Asp to Gly (614)	S
28881	GGG	AAC	Missense variant	aGGGga/aAACga	Arg/Gly to Lys/Arg (203)	N

a Lowercase letters indicate homology of nucleotides, and uppercase letters indicate nucleotide positions with changes. NA, not applicable.

Additional epidemiological and clinical features of this case of COVID-19 were reported in references 1 and 3.

### Data availability.

This sequence has been deposited in GenBank under the accession number MT318827. The accession numbers for the Illumina NextSeq sequencing raw reads in the NCBI Sequence Read Archive (SRA) are PRJNA624231 (BioProject), SRR11517432 (SRA), and SAMN14572083 (BioSample).
